# Prediction prolonged mechanical ventilation in trauma patients of the intensive care unit according to initial medical factors: a machine learning approach

**DOI:** 10.1038/s41598-023-33159-2

**Published:** 2023-04-12

**Authors:** Mohebat Vali, Shahram Paydar, Mozhgan Seif, Golnar Sabetian, Ahmad Abujaber, Haleh Ghaem

**Affiliations:** 1 Student Research Committee, Shiraz University of Medical Sciences, Shiraz, Iran; 2 Trauma Research Center, Shahid Rajaee (Emtiaz) Trauma Hospital, Shiraz University of Medical Sciences, Shiraz, Iran; 3 Non-Communicable Diseases Research Center, Department of Epidemiology, School of Health, Shiraz University of Medical Sciences, Shiraz, Iran; 4 Anesthesiology and Critical Care Trauma Research Center, Shiraz University of Medical Sciences, Shiraz, Iran; 5 Nursing, Hamad Medical Corporation, Doha, Qatar

**Keywords:** Diseases, Trauma, Statistics

## Abstract

The goal of this study was to develop a predictive machine learning model to predict the risk of prolonged mechanical ventilation (PMV) in patients admitted to the intensive care unit (ICU), with a focus on laboratory and Arterial Blood Gas (ABG) data. This retrospective cohort study included ICU patients admitted to Rajaei Hospital in Shiraz between 2016 and March 20, 2022. All adult patients requiring mechanical ventilation and seeking ICU admission had their data analyzed. Six models were created in this study using five machine learning models (PMV more than 3, 5, 7, 10, 14, and 23 days). Patients’ demographic characteristics, Apache II, laboratory information, ABG, and comorbidity were predictors. This study used Logistic regression (LR), artificial neural networks (ANN), support vector machines (SVM), random forest (RF), and C.5 decision tree (C.5 DT) to predict PMV. The study enrolled 1138 eligible patients, excluding brain-dead patients and those without mechanical ventilation or a tracheostomy. The model PMV > 14 days showed the best performance (Accuracy: 83.63–98.54). The essential ABG variables in our two optimal models (artificial neural network and decision tree) in the PMV > 14 models include FiO_2_, paCO_2_, and paO_2_. This study provides evidence that machine learning methods outperform traditional methods and offer a perspective for achieving a consensus definition of PMV. It also introduces ABG and laboratory information as the two most important variables for predicting PMV. Therefore, there is significant value in deploying such models in clinical practice and making them accessible to clinicians to support their decision-making.

## Introduction

About 30% of severely sick patients need prolonged mechanical ventilation (PMV)^1^. Since there is no specific definition for long-term mechanical ventilation, it is not possible to accurately evaluate these patients. But based on a previous study, the prevalence of long-term mechanical ventilation was estimated at 7.4 per 100,000 people^2^. A tracheostomy is finally performed on 10% of patients who require at least 3 days of artificial ventilation^3^. In addition, while MV is a life-saving procedure, it comes with a host of risks, including death, ventilator-associated pneumonia (VAP), ventilator-associated lung damage, and prolonged hospitalization^4,5^. Also, this topic can be effective with other variables, including the requirements of the patient's rights from the patients' point of view^6^. These risks are heightened with PMV^1,5^. As a result, predicting patients at risk of PMV is critical in assisting clinicians in developing unique care plans to reduce the risk of PMV^7–10^. Because, the policy makers and executive managers of the health system in Iran must prepare a comprehensive strategic plan for the improvement of hospitals for a proper, timely and up-to-date response^11^. However, the timing of tracheostomy remains controversial^12,13^.

Many studies were conducted to identify predictors of PMV. However, determining a set of crucial predictors remains challenging due to differences in the clinical characteristics of patients and clinical settings^14^. Also, one of the reasons for the difficulty of PMV studies is the lack of a consensus definition. Some believe that the ventilation PMV is more than 7 days^7,9,10,15^, some say 10 days^16^, some say 14 days^8,15^, and some say 21 days^17–19^. Also, as previously stated, most of the previous studies aimed at predicting PMV using multivariate techniques, particularly logistic regression, and had low to moderate accuracy^7,10^. Implementing machine learning to predict PMV has a relatively higher performance than conventional prediction models^7^. Also, in a previous study^20^, less emphasis was placed on laboratory data and Arterial Blood Gas. Given that the timing of tracheostomy is controversial, there is also no consensus definition for PMV, we decided to investigate the predictive performance of machine learning models in this study. Therefore, this study aims to use supervised machine learning to predict PMV time and tracheostomy time in ICU patients. Therefore, we are trying to determine a specific definition for PMV and specify the important and influencing variables.

## Methods

### Study design and patients

This retrospective cohort study included all patients that were admitted to the ICU of Iran’s Shahid Rajaei Hospital (the largest trauma center in Shiraz) between January 1, 2016 and March 20, 2022. We provided the data to ensure its quality and to complete patient information from three different sources in the hospital. These sources include Shiraz Intensive Care Intelligent Registry Data (in collaboration with the Anesthesia and Intensive Care Research Center with the Australian and New Zealand Intensive Care Association), the patient’s records in the hospital medical records, and the extracted patient information at the Trauma Research Center. Then we merged the information based on the patients’ file numbers.

**Figure 1:**
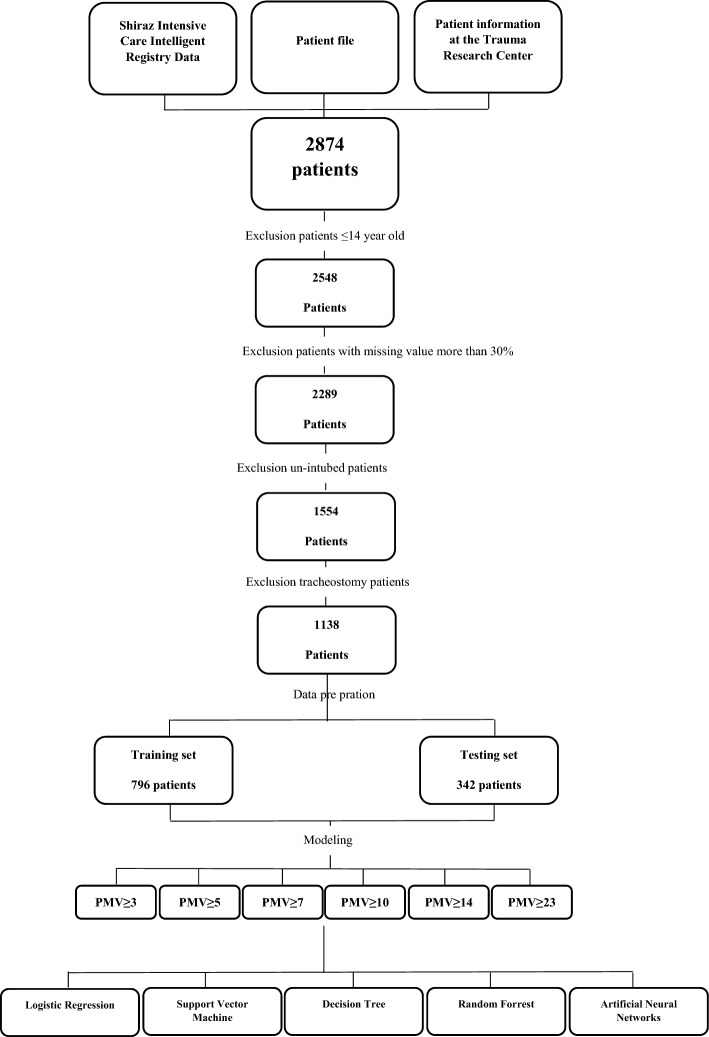
Study execution process.

Trauma patients over 14 years of age and patients who underwent intubation on the spot by emergency medical personnel or in the hospital following injury within the first 24 h were included in the study. Exclusion criteria for patients included (Fig. 1).

1. Patients diagnosed with brain death,
2. Lack of data at the beginning of hospital admission,
3. Transfering the patient to another hospital, and
4. Perform a tracheostomy and un-intubated patients.

Shiraz University of Medical Sciences ethics committee approved this study (IR.SUMS.SCHEANUT.REC.1400.006). We confirm that all research was performed in accordance with relevant guidelines and regulations, Also informed consent was obtained from their legal guardians according to the condition of the patients. We reported the findings per STROBE guidelines for strengthening observational studies (Supplementary Table S1).

### Data collection

We collected and recorded data on the following.

1. demographics, head trauma, or multiple trauma diagnosis,
2. Apache II and comorbidities (including Immune Disease, AIDS, Leukaemia Myeloma, Metastases, Lymphoma, Hepatic failure, Cirrhosis, Chronic liver failure, Chronic respiratory, Chronic Cardiovascular, and Chronic Renal failure),
3. Vital signs (including temperature, heart rate, systolic and diastolic blood pressure, arterial pressure, and Respiratory rate),
4. Length of hospitalization and length of stay in the intensive care unit,
5. Laboratory data (including Sodium, Potassium, Bicarbonate, Creatinine, Urea, Glucose, Hematocrit, Hemoglobin, White Blood Cell Count, Platelets),
6. Arterial Blood Gas (ABG) (including FiO_2_, PaO_2_, PaCO_2_, and pH), and
7. Interventions administered during hospitalization (such as endotracheal intubation and duration, Renal Replacement Therapy, and Thrombolytic Therapy) until discharge or death.

Importantly, we also collected data on the use of Inotropes Vasopressor along with complications such as Acute Renal Failure, Delirium, and Pressure Injury.

### Primary and secondary outcomes, subgroup analyses, and definition of features

For this study, we defined six PMV sets based on various definitions in the literature.

1. Patients who underwent endotracheal intubation for more than 3 days compared to those who had mechanical ventilation for less than 3 days (set A),
2. PMV more than 5 days (set B),
3. PMV more than 7 days (set C),
4. PMV more than 10 days (set D),
5. PMV more than 14 days (set E), and
6. PMV more than 23 days (set F).

The duration of ICU and hospitalization were calculated from ICU and hospitalization, respectively. Also, we calculated the number of days the patient was connected to the ventilator via an endotracheal tube.

Comorbidity was defined as the presence or absence of Immune Disease, AIDS, Leukemia, Myeloma, Metastases, Lymphoma, Hepatic failure, Cirrhosis, Chronic liver failure, Chronic respiratory, Chronic Cardiovascular, and Chronic renal failure.

The results of arterial blood gases were recorded in the ICU in the first 24 h based on blood gases. ABG values were used one hour before admission if arterial blood gases were not tested within the first 24 h of ICU admission. We also used the conversion table to convert oxygen to tail oxygen concentration.

We used the lowest level of consciousness in the first 24 h of the ICU to calculate the level of consciousness in the absence of sedatives, relaxants, or neuromuscular blocking drugs. In the case of sedatives, we also recorded the level of consciousness before administration. It is worth mentioning that the level of consciousness before receiving a sedative is not always the lowest level of consciousness. However, the level of consciousness is recorded before taking the sedative. GCS scoring was done in four sections and then added together.

1. Eye: open spontaneously = 4, open to voice = 3, open to pain = 2, do not open = 1;
2. Motor: obeys commands = 6, localises = 5, flexion withdrawal = 4, decorticate flexion = 3, extends = 2, nil = 1;
3. Verbal: orientated = 5, confused = 4, inappropriate words = 3, incomprehensible sounds = 2, no response = 1;
4. Verbal intubated: appears orientated = 5, ability to converse in doubt = 3, unresponsive = 1.

In the first 24 h of hospitalization in the ICU, patients’ physiological data, including vital signs and laboratory data, were recorded as their maximum, minimum, mean, and suffering. The values from one hour before admission to the ICU were used if the mentioned items were absent within the first 24 h.

### Statistical analysis

Several supervised machine learning methods were used to compare their performance with previous studies and to find a model characterized by optimum performance and maximum applicability to support clinical decision-making. We chose random forest (RF), logistic regression (LR), decision tree C.5 (C.5 DT), artificial neural networks (ANN), as well as support vector machines (SVM) so as to present the baseline comparative performance. Hyper-parameter values for each algorithm are given in Table S2. Also, we used RapidMiner Studio 9.10.008 and SPSS modeler ver 18.0 for analysis. Due to the imbalance of the classes, we used the SMOTE sampling method to balance them. Then, we divided the collected data into a testing set (30%) and a training set (70%) to avoid over-fitting and validate model performance. Given that the accuracy criterion alone is inadequate for assessing the overall performance of the model, besides the accuracy, AUC, sensitivity, Negative Predictive Value (NPV), F-score, and specificity were also considered. The F1 criterion, which is a balanced combination between precision and accuracy, is applicable in cases in which the cost of a false negative and a false positive differ. The same accuracy criterion can be used if the cost of False Negative and False Positive is nearly the same. However, if the data is unevenly distributed across classes (for example, 90% patients and 10% healthy), it is preferable to use the accuracy, recall, or F1 criteria (F1 score = 2*(Precision*Recall)/Precision + Recall).

We also selected the most important variables in our best model using the Brute Force algorithm and the importance of each variable was reported based on the gain ratio (Table S3).

#### Logistic regression (LR)

Logistic regression is one of the popular techniques used for the prediction of binary, multinomial, or ordinal outcomes^21^. This study uses stepwise LR (backward stepwise method based on likelihood) to control for confounding variables and also calculate independent risk factors for PMV after ICU admission.

#### Random forest (RF)

Random forest is a robust supervised machine learning method commonly employed to solve classification-related problems^21,22^. It has been demonstrated that RF is more accurate than other methods of machine learning. This accuracy is ascribable to the fact that random forest employs bootstrap for forest growth of unrelated trees characterized by high randomness in feature selection. This helps to reduce errors significantly^23^.

#### Support vector machine (SVM)

Support vector machine is a robust classification machine learning algorithm applied to linear and nonlinear data sets^24^. It is critical to determine which core function best reaches the optimal cloud page that separates classes when using SVM for classification purposes^25^. Also, this study used the radial basis function (RBF) to provide better predictive efficiency in the initial evaluation.

#### Artificial neural networks (ANNs)

Artificial neural networks are machine learning methods for pattern recognition and classification^24^. Researchers view artificial neural networks as an analytical model of the black box. Their undeniable capability of supporting clinical practice via interaction with evidence-based medicine is indisputable^26^. The present investigation employs ANN Multilayer Perception (MLP) because it had a better performance than the radial basis function (RBF) in the initial analysis.

The present investigation used a standard feed-forward neural network featuring three layers: a hidden layer, an output layer, and an input layer. A multilayer perception network is a novel tool for creating specialized layered feed-forward networks. These two layers are responsible for connecting the network to the outer world. A typical multilayer perception contains one or more layers of neurons. Because these layers are inaccessible directly, they are known as hidden neurons^27^. Hidden neurons are responsible for extracting the critical features from the input data. By partitioning data into separate experimental and training datasets to avoid overfitting, the neural network is typically optimized. The training process will continue until the error is reduced^28^. When utilized for classification, artificial neural networks are viewed as a collection of interconnected output/input units, each of which has its own associated weight. This value indicates the connection strength between the units^29^.

#### C.5 decision tree (DT)

The C.5 decision tree classification data mining algorithm replaces the C.4.5 decision tree classification algorithm of data mining. The DT is a “classification algorithm” where every single non-leaf node represents an experiment on one of the properties of the input items. “Every single branch corresponds to a test outcome, while each single leaf node represents a class prediction.”^30^. Decision trees are logical, robust, and easy to understand and interpret classification algorithms^31^.

### Ethics approval and consent to participate

The Ethics Committee approved this study at Shiraz University of Medical Sciences (IR.SUMS.SCHEANUT.REC.1400.006). Informed consent was obtained from all subjects or their legal guardians to use their data for research.

## Results

### Data mining algorithms’ performance

Table 1 shows the performance evaluation criteria for the five machine learning techniques on the test data partition. All models achieved accuracy (61.11–85.27). Random Forrest is the preferred model for deployment because it has a higher discriminating power (AUC = 0.821), which is critical for the classification function. In the set of models, logistic regression showed better or equal performance with Support Vector Machines and Artificial Neural Networks. When the discrimination power between the three sets is compared, set E, which defines PMV as more than 14 days, outperforms other groups, with AUC ranging from 83.70 to 90.20. This value demonstrates that the detection power and accuracy were optimal when the PMV was greater than 14 days.

**Table 1:** Performance of the prediction models.

Models	Accuracy (%)	Area under the curve	Precision (%)	Negative predictive value (%)	Sensitivity (%)	Specificity (%)	F1 score (%)
Model 1: PMV > 3 days							
Logistic regression	69.30	76.30	67.97	70.85	73.02	65.58	70.40
Support vector machine	70.70	77.80	69.60	71.92	73.49	67.91	71.49
Random forrest	76.90	82.10	89.60	70.37	60.87	92.93	72.49
Artificial neural networks	61.68	65.80	61.26	62.15	63.59	59.78	62.40
Decision tree	76.63	76.20	93.75	69.14	57.07	96.20	70.95
Model 2: PMV > 5 days							
Logistic regression	71.11	78.00	70.21	72.09	73.33	68.89	71.74
Support vector machine	73.56	81.00	75.24	72.08	70.22	76.89	72.64
Random forrest	81.56	88.40	93.29	74.83	68.00	95.11	78.66
Artificial neural networks	61.11	65.50	61.68	60.59	58.67	63.56	60.14
Decision tree	81.33	80.90	95.48	73.90	65.78	96.89	77.89
Model 3: PMV > 7 days							
Logistic regression	69.84	76.00	68.94	70.83	72.22	67.46	70.54
Support vector machine	75.20	81.50	74.71	75.71	76.19	74.21	75.44
Random forrest	80.36	89.10	92.74	73.54	65.87	94.84	77.03
Artificial neural networks	80.75	88.1	83.26	78.60	76.98	84.52	80.00
Decision tree	78.77	77.20	95.03	71.14	60.71	96.83	74.09
Model 4: PMV > 10 days							
Logistic regression	76.00	82.70	75.27	76.78	77.45	74.55	76.34
Support vector machine	85.27	88.90	89.11	82.12	80.36	90.18	84.51
Random forrest	84.55	91.70	91.67	79.50	76.00	93.09	83.10
Artificial neural networks	71.09	79.30	72.66	69.73	67.64	74.55	70.06
Decision tree	84.36	85.30	90.91	79.62	76.36	92.36	83.00
Model 5: PMV > 14 days							
Logistic regression	77.80	83.70	77.89	77.70	77.62	77.97	77.76
Support vector machine	82.52	85.80	87.80	78.53	75.52	89.51	81.20
Random forrest	81.99	89.70	90.31	76.52	71.68	92.31	79.92
Artificial neural networks	85.14	90.2	87.36	83.17	82.17	88.11	84.68
Decision tree	82.69	82.60	92.31	76.64	71.33	94.06	80.47
Model 6: PMV > 23 days							
Logistic regression	79.86	85.40	81.16	78.67	77.78	81.94	79.43
Support vector machine	84.55	88.10	91.29	79.70	76.39	92.71	83.18
Random forrest	84.90	91.10	94.67	78.63	73.96	95.83	83.04
Artificial neural networks	73.44	78.7	76.89	70.77	67.01	79.86	71.61
Decision tree	84.20	83.70	93.39	78.22	73.61	94.79	82.33

*PMV* prolonged mechanical ventilation.

### Characteristics of participants

Data characteristics of participants are shown in Table 2. In this study, 1138 ICU patients with a mean age (years) of 44.50 ± 21.42 (Max: 100 and Min = 18) participated. Of all participants, 929 (81.6%) were male. The median hospitalization time (day) for all participants was 29.50 (8, 47). APACHE II Risk (%) was higher in MV for more than 14 days (P.value = 0.026). ICU stay in PMV ≥ 14 was longer (7 vs. 49, P.value** ≤ **0.001).

**Table 2:** Characteristics participants and logestic regression (univariate and multivarieate) for PMV > 14.

Variable	PMV		Total (n = 1138)	P.value*	Crude OR 95% CI	Adjusted OR 95% CI
	< 14 days	≥ 14 days				
Age (year)	44.25 ± 21.60	45.76 ± 20.46	44.50 ± 21.42	0.381	1.003 (0.99, 1.01)	–
Day hospitan (IQR)	15 (7, 42)	48 (37, 65.25)	29.50 (8, 47)	**≤ 0.001**	1.004 (1.003, 1.006)	1.008 (1.006, 1.010)
BMI (kg/m^2^)	25.00 ± 3.70	25.33 ± 5.18	25.05 ± 3.98	0.301	1.019 (0.98, 1.057)	–
APACHE II Risk (%)	32.97 ± 20.58	36.68 ± 21.17	33.57 ± 20.72	**0.026**	1.008 (1.001, 1.016)	–
ICU day (IQR)	7 (3.00, 16.75)	49 (40.00, 55.25)	9 (3, 40)	**≤ 0.001**	1.008 (1.006, 1.010)	–
Mean temperature (°C)	37.46 ± 0.81	37.66 ± 3.03	37.49 ± 1.43	0.172	1.071 (0.971, 1.182)	–
Mean HR (bpm)	94.13 ± 18.51	95.99 ± 19.42	94.41 ± 18.67	0.214	1.005 (0.997, 1.014)	–
Mean systolic (mmhg)	121.68 ± 13.84	121.40 ± 14.18	121.63 ± 13.89	**0.004**	1.507 (1.139, 1.995)	–
Mean diastolic (mmhg)	72.98 ± 10.30	73.99 ± 10.80	73.15 ± 10.38	**0.003**	2.339 (1.334, 4.100)	–
Mean arterial pressure (mmhg)	88.89 ± 10.19	89.19 ± 10.85	88.94 ± 10.29	**0.003**	0.286 (0.123, 0.662)	–
Mean respiratory rate (bpm)	17.69 ± 3.71	17.93 ± 3.54	17.73 ± 3.68	0.280	1.022 (0.982, 1.064)	–
Sodium (mmol/L)	141.18 ± 6.65	141.16 ± 11.44	141.18 ± 7.64	0.181	1.016 (0.993, 1.040)	–
Potassium (mmol/L)	4.39 ± 0.71	4.54 ± 1.57	4.42 ± 0.97	0.613	1.058 (0.851, 1.315)	–
Bicarbonate (mmol/L)	23.07 ± 4.039	22.59 ± 4.00	23.00 ± 4.03	0.479	0.985 (0.945, 1.027)	–
Creatinine (IQR) (mg/dl)	1.10 (0.90, 1.36)	1.12 (0.95, 1.40)	1.10 (0.90, 1.37)	0.371	0.986 (0.957, 1.016)	–
Urine output (ml/day)	3007.73 ± 834.61	2877.43 ± 773.14	2986.43 ± 825.95	0.0694	0.9998 (0.9996, 1.0000)	–
Urea (mg/dl)	18.13 ± 13.78	19.45 ± 12.00	18.35 ± 13.51	0.759	1.002 (0.991, 1.013)	–
Glucose (mg/dl)	159.60 ± 69.20	165.44 ± 67.77	160.56 ± 68.97	0.544	1.001 (0.998, 1.003)	–
Hematocrit (IQR) (%)	0.37 (0.33, 0.41)	0.36 (0.33, 0.41)	0.37 (0.33, 0.41)	0.602	1.76 (0.21, 14.56)	–
Hemoglobin (g/dl)	12.56 ± 2.16	12.56 ± 2.38	12.56 ± 2.20	0.258	0.903 (0.757, 1.078)	–
White blood cell count (/mm^3^)	14.62 ± 6.01	16.57 ± 19.27	14.93 ± 9.55	0.146	1.014 (0.995, 1.032)	–
Platelets (10^3^/µL)	210.09 ± 80.96	202.26 ± 78.07	208.80 ± 80.52	0.258	0.999 (0.996, 1.001)	–
FiO_2_	0.41 ± 0.06	0.43 ± 0.08	0.41 ± 0.06	**0.010**	12.66 (1.81, 88.36)	9.477 (1.246, 72.091)
paO_2_	141.67 ± 58.41	146.08 ± 69.50	142.39 ± 60.35	0.399	1.001 (0.999, 1.004)	–
paCO_2_	36.75 ± 7.27	34.78 ± 7.41	36.42 ± 7.32	**0.001**	0.962 (0.939, 0.985)	0.966 (0.944, 0.989)
pH	7.34 ± 0.08	7.30 ± 0.47	7.33 ± 0.20	0.751	0.747 (0.124, 4.510)	–
GCS	8.81 ± 4.62	8.16 ± 4.59	8.71 ± 6.62	0.138	0.974 (0.940, 1.009)	–
Sex						
Male	786 (84.6%)	143 (15.4%)	929 (81.6%)	0.067	1.42 (0.97, 2.08)	–
Female	166 (79.4%)	43 (20.6%)	209 (18.4%)			
Known comorbidities						
Yes	70 (7.4%)	10 (5.4%)	80 (7.0%)	0.337	0.716 (0.362, 1.416)	
No	882 (92.6)	176 (94.6%)	1058 (93.0%)			
Diagnosis						
Head trauma	525 (55.1%)	115 (61.8%)	640 (56.2%)	Ref	Ref	–
Multiple trauma	323 (33.9%)	53 (28.5%)	376 (33.0%)	0.392	1.266 (0.738, 2.171)	
Other	104 (10.9%)	18 (9.7%)	122 (10.7%)	0.957	0.948 (0.532, 1.691)	
Renal replacement therapy						
Yes	31 (3.3%)	9 (4.8%)	40 (3.5%)	0.287	1.511 (0.707, 3.228)	–
No	921 (96.7%)	177 (95.2%)	1098 (96.5%)			
Inotropes vasopressor						
Yes	150 (15.8%)	53 (28.5%)	203 (17.8%)	**≤ 0.001**	2.210 (1.524, 3.206)	1.959 (1.339, 2.867)
No	802 (84.2%)	133 (71.5%)	935 (82.2%)			
Delirium						
Yes	14 (1.5%)	2 (1.1%)	16 (1.4%)	0.394	0.515 (0.112, 2.375)	–
No	938 (98.5%)	184 (98.9%)	1122 (98.6%)			
Pressure injury						
Yes	53 (5.6%)	11 (5.9%)	64 (5.6%)	0.976	0.990 (0.498, 1.966)	–
No	899 (94.4%)	175 (94.1%)	1074 (94.4%)			
Thrombolytic therapy						
Yes	168 (17.6%)	39 (21.0%)	207 (18.2%)	0.357	1.205 (0.810, 1.794)	–
No	784 (82.4%)	147 (79.0%)	931 (81.8%)			
Acute renal failure status						
Yes	20 (2.1%)	4 (2.2%)	24 (2.1%)	0.514	0.690 (0.226, 2.101)	–
No	932 (97.9%)	181 (97.3%)	1113 (97.8%)			

*PMV* prolonged mechanical ventilation, *OR* odds ratio, *IQR* interquartile range, *FiO*_*2*_ fraction of inspired oxygen, *PaO*_*2*_ partial pressure of oxygen, *PaCO*_*2*_ partial pressure of carbon dioxide, *pH* potential of hydrogen, *GCS* Glasgow Coma Scale.

Significant values are in [bold].

*P.value ≤ 0.05.

### Logistic regression (LR)

The relationship between hospitalization days, APACHE II Risk, ICU days, Mean systolic, Mean diastolic, Mean arterial pressure, Urine Output, FiO_2_, PaCO_2_, Inotropes Vasopressor, and PMV ≥ 14 days was investigated using logistic regression. PMV ≥ 14 days odds of occurring increased by 1.08 times (95% CI (1.006, 1.010) for a one-unit increase in the number of days in the hospital. It also increased by 9.47 times (95% CI (1.246, 72.091) for a one-unit increase in FiO_2_ and by 0.966 times (95% CI (0.944, 0.989) for a one-unit increase in PaCO_2_. The odds of PMV ≥ 14 days increased by 1.959 times (95% CI (1.339, 2.867)) for patients who did not use Inotropes Vasopressor compared to used Inotropes Vasopressor.

### Decision tree

**Figure 2:**
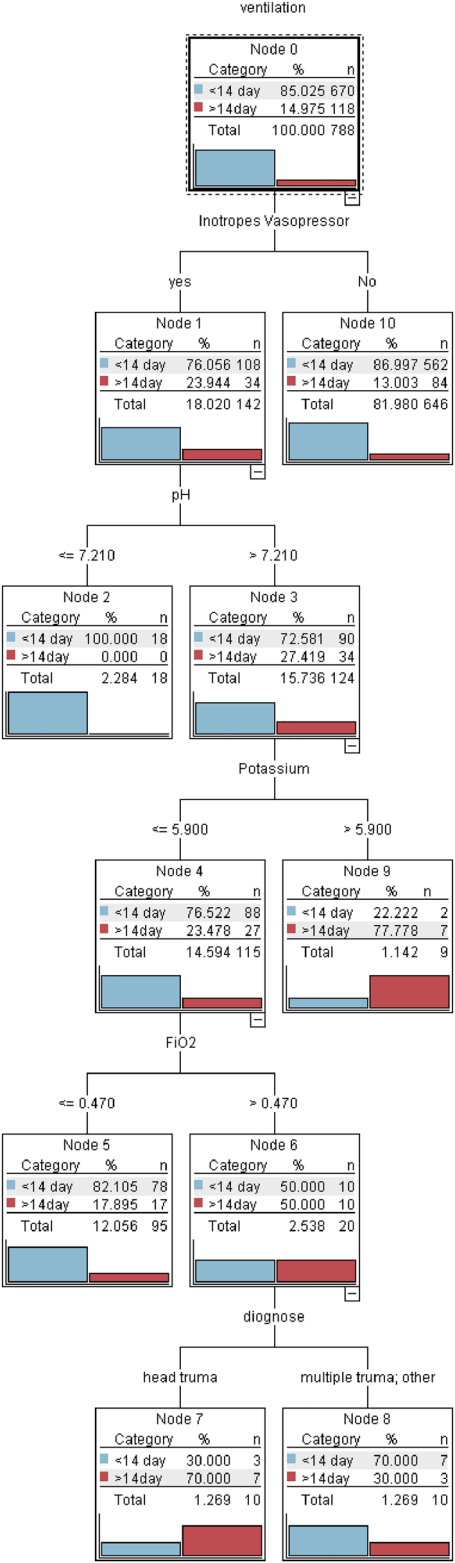
Decision tree for predicting prolonged mechanical ventilation in the intensive care unit. potential of hydrogen (pH), Fraction of inspired oxygen (FiO_2_).

**Figure 3:**
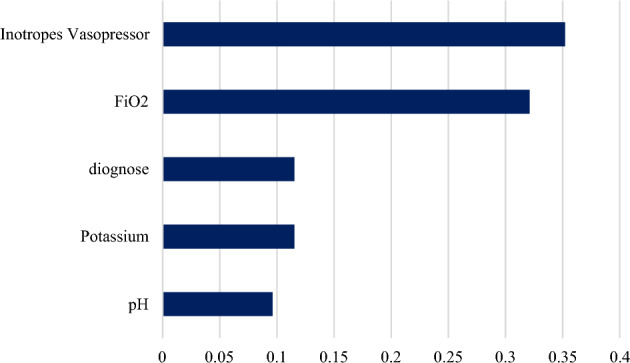
Predictor importance chart for decision tree, potential of hydrogen (pH), Fraction of inspired oxygen (FiO_2_).

As shown in Fig. 2, the variable of the initial split in the DT model was inotropes vasopressor. The second split’s variable was identified as pH, with an optimal cut-off value of 7.21. This value was recognized among the patients with inotropes vasopressor = yes. On the third split, Potassium, with an optimal cut-off value of 5.9, and FiO_2_, with an optimal cut-off value of 0.470, were identified. The diagnosis was then identified as the final split. According to the DT analysis, the variables in the order of importance were: inotropes vasopressor, FiO_2_, diagnose, Potassium, and pH. Figure 3 depicts the relative importance of the variables.

### Random forrest (RF)

The random forest model’s test classification accuracy was 81.99%, and its F1 score was 79.92%. PaCO_2_, pH, and hemoglobin were the three most important predictors of prolonged mechanical ventilation.

### Support vector machines (SVM)

We ran the SVM model with radial and gamma kernels of 1.0, kernel cache of 200, C with zero value and convergence epsilon of 0.001, and maximum iterations of 100,000. The model’s accuracy was 82.52. Classification error and F1 scores were 17.48% and 81.20%, respectively (Table 1). Markedly, the total number of Support Vectors and Bias (offset) were 1332 and -0.567, respectively. The most important predictors of prolonged mechanical ventilation were: diagnosis, gender, age, inotropes vasopressor, APACHE II, PaCO_2_, PaO_2_, Glucose, Potassium, and systolic pressure.

### Artificial neural networks (ANN)

**Figure 4:**
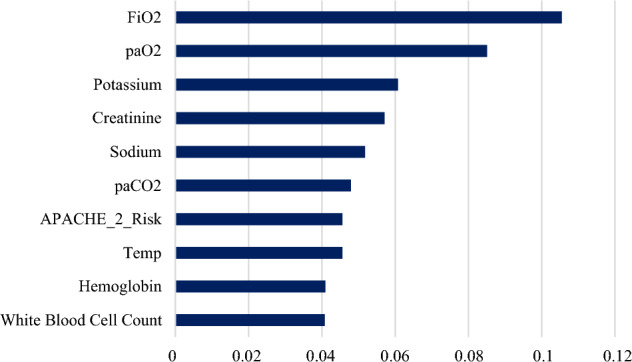
Predictor importance chart for Artificial Neural Networks partial pressure of carbon dioxide (PaCO_2_), Fraction of inspired oxygen (FiO_2_), partial pressure of oxygen in the arterial blood (PaO_2_), Temperature (Temp).

**Figure 5:**
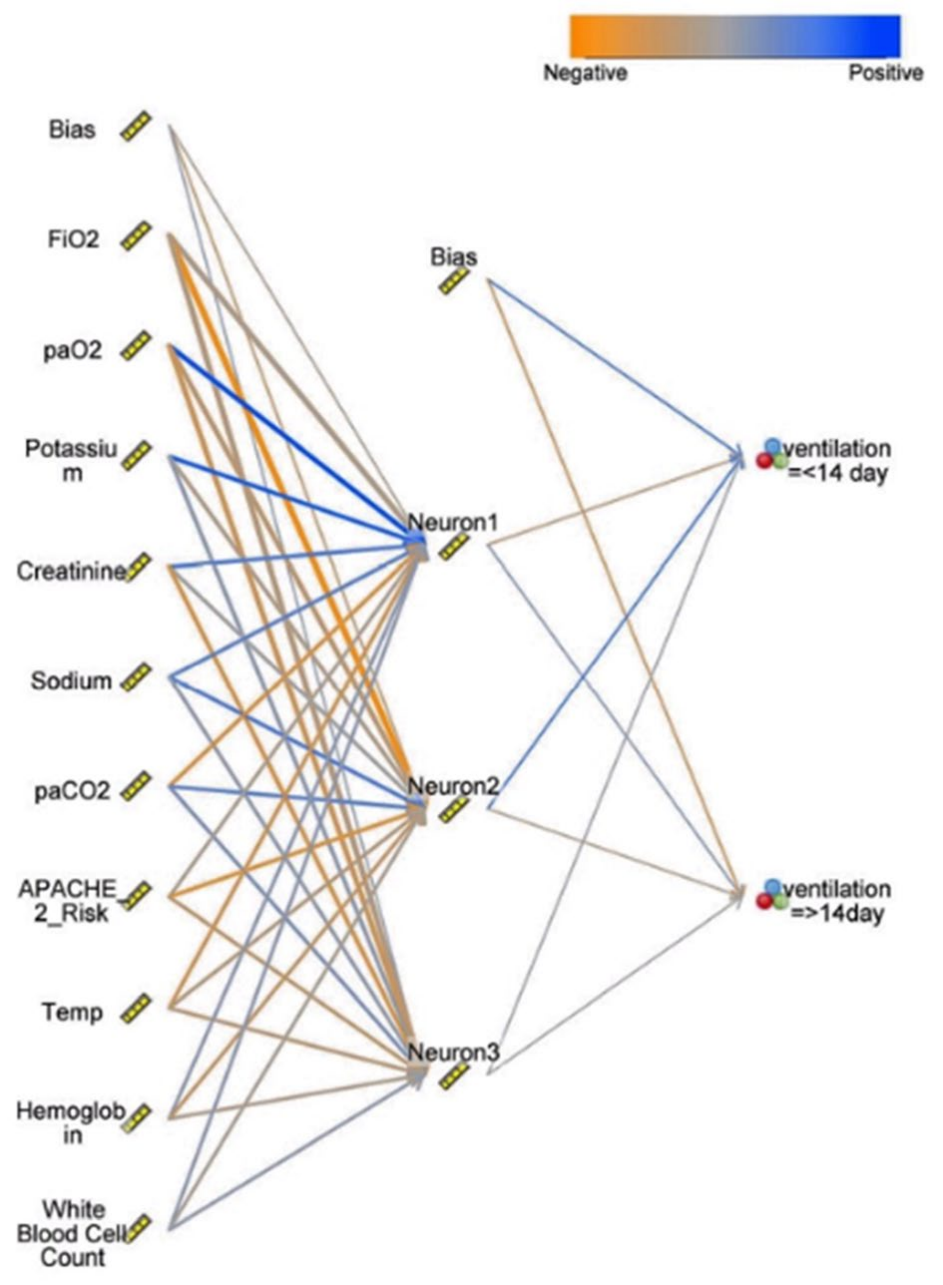
Schematic of the artificial neural network (ANN) constructed here. 50 input variables were compared.

The network was trained with a training cycle of 200 and a momentum of 0.9 using RBF transfer functions with upper and lower limits of 1 and − 1 and a constant learning rate of 0.01. The accuracy of ANN was 85.14%. The classification error was 14.86% (Table 1). The performance of ANN is shown in Table 1. FiO_2_, PaO_2_, Potassium, Creatinine, Sodium, PaCO_2_, APACHE II, Temperature, Hemoglobin, and White blood cell are the essential variables in this model (Figs. 4 and 5). ROC curve for comparing all 5 machine learning algorithms in 6 models used under stratified tenfold cross-validation is given in Fig. S1.

## Discussion

The current study was designed to investigate predicting PMV. We discovered the following results in this study. First, the most critical variables in predicting PMV were vasopressor inotropes, FiO_2_, PaO_2_/FiO_2_, PaCO_2_, and APACHE II. Second, most models found Potassium to be the most crucial variable in predicting PMV. Finally, based on modeling accuracy and output ROC plots, we chose 14 days as the cut-off point for distinguishing PMV from non-PMV. As a result, when PMV is predictable, it is critical to plan tracheostomy early and possibly transfer the patient to the most appropriate institution for PMV and its complications. Furthermore, the use of supervised machine learning techniques especially ANN resulted in moderate to high overall performance across six ensembles, according to this study.

Utilizing vasopressor inotropes was one of the current study’s most significant variables in predicting PMV. Fluid resuscitation and vasoactive therapy are critical in managing hypotensive patients to support organ perfusion^32–34^. Current guidelines from the 2016 Surviving Sepsis Campaign (SSC) recommend early initiation of vasopressors targeting mean arterial pressure ≥ 65 mmHg^35^. Guidelines specify that evaluating the need for vasopressor therapy starts when there is persistent hemodynamic instability despite fluid resuscitation. The SSC guidelines are not specific about when to initiate vasopressors. However, recent studies have shown that delaying the initiation of vasopressors is associated with a higher mortality rate, fewer vasopressor-free days, and a longer time to achieve target mean arterial pressure^36,37^. Following the patient’s hemodynamic instability, PMV occurs in those who have not used vasopressor inotropes.

FiO_2_ and PaO_2_ was one of our essential variables and predicted PMV for us. PaO_2_/FiO_2_ on the third day of intubation was also found to be a predictor of PMV by Sellers et al.^38^. Long-term excessive FiO_2_ exposure may be associated with pulmonary function deterioration in a dose–response manner. Excess FiO_2_ exposure was not associated with mortality in one study^39^. However, in another large retrospective study of mechanically ventilated ICU patients in the Netherlands, high FiO_2_ values during the ICU stay were associated with hospital mortality^40^. In the same study, high FiO_2_ in the first 24 h of hospitalization was linearly related to in-hospital mortality rates of high and low PaO_2_ values^40^. Long-term exposure to FiO_2_ has been shown in previous studies to have a linear correlation with a worsening oxygenation index. Furthermore, regardless of FiO_2_, the oxygenation index increased. This increase was linked to a longer length of mechanical ventilation and ICU stay. The Oxygenation index, combining airway pressure and oxygenation, is a reliable predictor of worsening lung function, particularly in patients with acute lung injury^41–43^. Nash et al. performed the first detailed pathological examination of pulmonary changes following exposure to higher oxygen concentrations and treatment duration. They observed significant pathological changes in interstitial edema with prolonged treatment that progressed to fibrosis^44^.

Another of the principal variables for PMV prediction is PaCO_2_. In another study^45^, PaCO_2_ was the only predictor of the need for PMV as a component of arterial blood gas analysis. However, it only predicted the need for PMV in two studies of patients admitted to the ICU for various reasons^46,47^. Interestingly, while low bicarbonate levels did not affect the likelihood of experiencing PMV^48^, a pH less than 7.25 significantly predicted the need for PMV for more than 14 days^48,49^. Our study is similar to the results of this study which shows the importance of pH value after PaCO_2_. Our study discovered a pH cut-off point of 7.21, consistent with previous research^48,49^.

APACHE II is the most widely used tool for predicting the result worldwide. Almost all staff, including doctors, nurses, physiotherapists, and social workers, are well-versed in it. Also, there is no single predictor of clinical outcome in PMV patients, as it is determined by a combination of respiratory and non-respiratory factors^50^. As a result, one of our goals was to assess the APACHE II scoring system’s ability to predict PMV. We discovered that APACHE II has high predictive power for PMV. This finding differs from the previous study^45^, which found that ICU admission severity scores did not have a high predictive power when using the statistical method. One plausible explanation is that these scores may lose predictive power in patients with extended hospital stays^51^. Likewise, Rojek-Jarmuła et al.^50^ discovered that in patients admitted to a weaning center for PMV, the APACHE II score could not predict successful freedom from mechanical ventilation or tracheostomy tube removal. However, predictive ability appears relatively better in studies with short PMV values^46^.

As a result, systematic reviews on the predictive ability of ICU scores for PMV are recommended as future collective evidence. APACHE II, on the other hand, has been validated by several researchers in predicting weaning success^52,53^. One reason could be that each center has different admission criteria and a different patient population regarding demographics, primary diagnoses, and comorbidities. Third, no single scoring system applies to all patients^50^. However, Safavi and Honarmand^54^ demonstrated that the APACHE III score might better predict the need for MV than the APACHE II, indicating the potential role of this new scoring system.

One of the crucial variables observed is the Potassium. According to our findings, Potassium is one of the laboratory data variables that can help predict PMV. In another study, Potassium disorder is one of the significant side effects that can lead to death, long-term mechanical ventilation (more than 21 days), and hospitalization (more than 36 days)^55^. Another study found that a patient’s Potassium level at the start of intensive care was strongly associated with the risk of death, even if it was slightly above the normal range^56^. In addition, Potassium level is mentioned as one factor indicating a higher risk of death in this group in another study conducted on Covid-19 patients hospitalized in the ICU^57^. Abnormal plasma Potassium levels may be a symptom of an acid–base imbalance in patients suffering from acute respiratory failure. Markedly, it can also result in cardiac arrhythmia, Brady arrhythmia, complete heart block, and circulatory arrest^56,58^. Previous research has examined the link between Potassium and mortality in the intensive care unit. According to their findings, Potassium mortality is independent of AKI, selective b1 blockade, or ACEi/ARB use. There was no correlation between Potassium and mortality in patients with K > 5.5 mEq/l who received blood transfusions and Potassium supplements before Potassium administration. The Potassium-mortality relationship was not adjusted for the presence of a K-promoting drug in its entirety. A C1 mEq/l decrease in Potassium within 48 h of the start of intensive care eliminates the Potassium-mortality association. The mechanism underlying the link between Potassium and mortality is unknown. The most apparent effect of hyperkalemia is a decrease in the myocardium’s resting membrane potential. This effect leads to a reduction in the speed of myocardial cell conduction and an increase in the repolarization rate^59^.

One of our hypotheses is that PMV may have contributed to increased mortality in patients with Potassium deficiencies. However, the degree of hyperkalemia is not associated with the risk of life-threatening arrhythmias in general^60^. Nonetheless, the progression of arrhythmias in hyperkalemia from benign to fatal is unpredictable^61^. Also, Potassium concentration may be an indicator of disease severity or may be related to an unmeasured patient factor that may be a cause of PMV in and of itself.

Finally, Set E outperformed the other sets regarding stability and discrimination power, with an average AUC of 87.075 compared to the other sets’ average AUC. This finding is significant because the optimal predictive performance is achieved when PMV is defined as more than 14 days, which is the optimal period for primary tracheostomy. However, unlike the previous study^15^, the current work did not record a better performance of machine learning than traditional forecasting techniques. It achieved nearly the same level of accuracy and precision as machine learning. However, machine learning, particularly ANN and decision tree methods, outperformed the traditional logistic regression method in terms of accuracy. Accordingly, machine learning techniques outperform conventional analytical techniques and can provide clinicians with more support for higher-quality decision-making, which improves patient treatment outcomes.

This study has its strengths; however, several potential limitations are concerning. First, the data used comes from the province’s largest trauma center. We used three data sources to ensure the information’s accuracy. However, our analysis is based on single-center retrospective data. Our findings have limited external validity and cannot be generalized to a larger international population.

Second, to the best of our ability, we attempted to track the patients using the case codes. Furthermore, we tried to analyze six different models using five machine learning methods that were thought to be the most powerful statistical methods available.

Further, the laboratory and ABG data, which have received less attention in previous studies, are regarded as one of the study’s strengths because these two variables can be measured and investigated relatively simply, allowing doctors to make decisions based on them. However, we did not include comprehensive data on sedation protocols and spontaneous breathing tests. Finally, we lack follow-up data on hospital readmission rates, MV reestablishment, and long-term mortality rates.

## Conclusion

This study found that ABG (particularly FiO_2_, PaO_2_, and PaCO_2_) and laboratory data (particularly Potassium) could assist physicians in predicting PMV. This study also demonstrated that a machine-learning approach could improve predictive power. However, improving data quality in registries or electronic medical records is more important than prediction, which helps enhance prediction quality. Furthermore, there is significant value in deploying such models in clinical practice and making them accessible to clinicians to support their decision-making. Also, like the results of the previous study^62^, the results of our study can help nurses working in the intensive care unit to make a better decision about when to perform tracheostomy and to be trained for it. We suggest that in future studies, attention should be paid to the measurements of variables over time in modeling, as well as other variables such as hospital readmission rates, MV reestablishment, and long-term mortality rates. Also, the use of multi-center data in future studies is suggested, which can control the difference of different treatment instructions to a great extent and increase the external validity of the study.

## Supplementary Information

Supplementary Information.

## Data Availability

The data that support the findings of this study are available from the corresponding author, [HGH], upon reasonable request.

## Abbreviations

PMV: Prolonged mechanical ventilation
ICU: Intensive care unit
ABG: Arterial blood gas
LR: Logistic regression
ANN: Artificial neural networks
SVM: Support vector machines
RF: Random forest
C.5 DT: C.5 decision tree
VAP: Ventilator-associated pneumonia
NPV: Negative predictive value
RBF: Radial basis function
MLP: Multilayer perception
SSC: Surviving sepsis campaign
OR: Odds ratio
IQR: Interquartile range
FiO_2_: Fraction of inspired oxygen
paO_2_: Partial pressure of oxygen
paCO_2_: Partial pressure of carbon dioxide
pH: Potential of hydrogen
GCS: Glasgow Coma Scale
Temp: Temperature
