# Author Correction: Prediction prolonged mechanical ventilation in trauma patients of the intensive care unit according to initial medical factors: a machine learning approach

**DOI:** 10.1038/s41598-023-37355-y

**Published:** 2023-06-23

**Authors:** Mohebat Vali, Shahram Paydar, Mozhgan Seif, Golnar Sabetian, Ahmad Abujaber, Haleh Ghaem

**Affiliations:** 1 Student Research Committee, Shiraz University of Medical Sciences, Shiraz, Iran; 2 Trauma Research Center, Shahid Rajaee (Emtiaz) Trauma Hospital, Shiraz University of Medical Sciences, Shiraz, Iran; 3 Non-Communicable Diseases Research Center, Department of Epidemiology, School of Health, Shiraz University of Medical Sciences, Shiraz, Iran; 4 Anesthesiology and Critical Care Trauma Research Center, Shiraz University of Medical Sciences, Shiraz, Iran; 5 Nursing, Hamad Medical Corporation, Doha, Qatar

Correction to: *Scientific Reports* 10.1038/s41598-023-33159-2, published online 12 April 2023

In the original version of this Article Mozhgan Seif was incorrectly affiliated with ‘Non-Communicable Research Center, Department of Epidemiology, School of Health, Shiraz University of Medical Sciences, Shiraz, Iran’. The correct Affiliation is listed below.

**Mozhgan Seif:**

Non-Communicable Diseases Research Center, Department of Epidemiology, School of Health, Shiraz University of Medical Sciences, Shiraz, Iran.

The original Article has been corrected.

